# Hypertension: A Continuing Public Healthcare Issue

**DOI:** 10.3390/ijms26010123

**Published:** 2024-12-26

**Authors:** Samaneh Goorani, Somaye Zangene, John D. Imig

**Affiliations:** 1 Department of Pharmaceutical Sciences, University of Arkansas for Medical Sciences, Little Rock, AR 72205, USA; 2 Faculty of Medicine, University of Tehran, Tehran 1416634793, Iran; so.zangene@ut.ac.ir

**Keywords:** blood pressure, anti-hypertensive, cardiovascular, lifestyle

## Abstract

Hypertension is a cardiovascular disease defined by an elevated systemic blood pressure. This devastating disease afflicts 30–40% of the adult population worldwide. The disease burden for hypertension is great, and it greatly increases the risk of cardiovascular morbidity and mortality. Unfortunately, there are a myriad of factors that result in an elevated blood pressure. These include genetic factors, a sedentary lifestyle, obesity, salt intake, aging, and stress. Although lifestyle modifications have had limited success, anti-hypertensive drugs have been moderately effective in lowering blood pressure. New approaches to control and treat hypertension include digital health tools and compounds that activate the angiotensin receptor type 2 (AT2), which can promote cardiovascular health. Nonetheless, research on hypertension and its management is vital for lessening the significant health and economic burden of this condition.

## 1. Introduction

Hypertension continues to be the most prevalent cardiovascular disease and mortality risk factor [1,2]. Since 2003, the World Health Organization’s Global Burden of Disease Study has identified hypertension as the preeminent worldwide risk factor contributing to both morbidity and mortality [3]. Even among those who are presumed to have well-controlled hypertension, less than one-third are safeguarded against subsequent strokes and heart attacks [4]. The WHO World Report on Blood Pressure, released in 2023, highlights the staggering statistics surrounding the prevalence of high blood pressure, noting that nearly 1.3 billion adults were estimated to be living with the condition by 2019. This report shows that only 54% of adults with high blood pressure have been diagnosed, only 42% are receiving treatment, and only 21% have their blood pressure adequately controlled [5]. The current state of practice is manifestly inadequate; an insufficient number of individuals who are at risk due to hypertension are being accurately diagnosed and effectively treated [6]. An additional factor contributing to the complexity of hypertension’s etiology is its multifactorial nature [7]. As a result, significant advancements are required in both population-wide and individual strategies for the prevention and management of hypertension [8]. Numerous clinical trials have yielded conclusive results that blood pressure reduction effectively mitigates cardiovascular morbidity and mortality across the board, including in high-risk normotensive individuals with hypertension of any severity [9]. Rapid globalization, unplanned urbanization, global trade, and agricultural policies are a few of these determinants that ultimately affect the capacity of a society or an individual to make healthy decisions, thereby contributing to the adverse effects on social and economic development in low- and middle-income countries [10]. Significant economic repercussions result from the necessity to reallocate limited resources to tertiary care and the loss of productive years of life [10]. We will focus here on a few influential issues that we believe have an impact on hypertensive cardiovascular disease, and this review focuses on the social circumstances of people’s lives.

## 2. Epidemiology of Hypertension

Previous studies have shown that the estimated global average blood pressure has remained stable or slightly decreased over the past four decades [11]. In contrast, the variability of regional changes in estimated mean blood pressure from 1975 to 2015 was higher. Overall, blood pressure decreased significantly in high-income countries, while it increased in low- and middle-income countries. Between 1975 and 2015, the age-standardized mean systolic and diastolic blood pressure decreased significantly in Western and Asia–Pacific regions for both men and women [12]. A decrease in systolic blood pressure was recorded, with a decrease of 2.4 mmHg per decade for men and 3.2 mmHg per decade for women [11]. Likewise, western regions with higher average incomes showed a reduction of about 1.5 mmHg per decade for men and 1.8 mmHg per decade for women [11,13].

Given the domains of epidemiology and pathogenesis, certain subgroups, including elderly women and blacks, possess unique attributes that necessitate further investigation [14]. The prevalence of hypertension is relatively lower in women and men up to the age of 45 years [15], it is almost similar between both sexes between the ages of 45 and 64 years, and it increases significantly in women over 65 years of age compared to men and younger people [16]. Most women (48.8% between 60 and 79 years and 63% after 80 years) have stage 2 hypertension (BP 160/100 mmHg) and are prescribed anti-hypertensive therapy [17]. Menopause is associated with complications, such as spontaneous ovarian failure, endothelial dysfunction, increased arterial stiffness, obesity, genetic factors, high total cholesterol, and low-density lipoprotein cholesterol levels with increased hypertension [18]. Likewise, it is challenging to provide an appropriate way to control hypertension for older women [19].

In the United States, the highest morbidity and mortality from hypertension are observed in African Americans, and they are less likely to achieve proper blood pressure control [20]. People of African descent have a more severe early-onset and uncontrolled form of hypertension, which is associated with the highest mortality from coronary artery disease, stroke, left ventricular hypertrophy, regurgitation heart failure, and chronic kidney disease [19,21] to the extent that one of the major factors in the disproportionately low life expectancy of African American men is hypertension. All of these complications occur at an average age of 70.0 years for African American women, 75.9 years for men, 76.8 years for Caucasian men, and 80.8 years for Caucasian women [17].

## 3. Disease Burden

It was estimated that between 2011 and 2015, the total economic loss attributable to cardiovascular disease in low- and middle-income countries was USD 3.7 trillion (2010), representing roughly half of the economic burden attributable to non-communicable diseases and 2% of the Gross Domestic Product (GDP) in low- and middle-income countries [22]. Attaining the global objective of a 25% reduction in cardiovascular disease mortality by 2025 might not be adequate in many low- and middle-income countries, especially in the most impoverished regions [22]. The direct and indirect costs of high blood pressure significantly strain healthcare systems. Direct costs related to hypertension include medical and hospital care costs. The average cost of hospital care for patients with high blood pressure can reach approximately USD 21,094 per year, which is a significant amount. On the other hand, indirect costs include economic losses due to reduced productivity, disability, and premature mortality associated with uncontrolled blood pressure [23].

There has been a surge in the burden of cardiovascular disease in sub-Saharan Africa, namely hypertension, renal disease, and heart failure. This epidemic in cardiovascular disease poses an additional burden on the already over-burdened healthcare systems. In these settings, hypertension is creating critical challenges for both national health systems and policy development that impede the development of a strategic plan to address the cardiovascular disease epidemic [10].

In China, hypertension, cardiovascular disease, and mortality are significant public health concerns [24]. It is critical to develop and implement anti-hypertensive intervention strategies that are practical, sustainable, and effective to reduce morbidity and mortality associated with hypertension [25]. It has been postulated that treating all cases of hypertension in China could prevent approximately 800,000 cardiovascular disease events annually in a cost-effective manner [26]. Programs for low-cost, essential anti-hypertensive medications must be established and maintained [26]. Additional benefits could result from the simplification of hypertension guidelines, the improvement of home blood pressure measurement, and the identification of individuals with uncontrolled hypertension [27]. To combat the morbidity and mortality associated with hypertension, a comprehensive strategy at the national and local levels will be required [28].

## 4. Cardiovascular Disease Risk

Cardiovascular disease is a disorder of the circulatory system and the heart. It is a collection of diverse diseases that frequently have atherosclerosis as their underlying cause of development [29]. Cardiovascular diseases are lifelong conditions characterized by modest progression and prolonged asymptomatic status. If present, symptoms typically manifest in advanced stages of the disease or as sudden death [30]. They have been the primary cause of premature mortality worldwide for years. Annually, 23.6 million individuals are projected to succumb to cardiovascular disease by 2030. A modest inclination exists towards reducing the incidence and mortality of cardiovascular disease in the northwestern and southern regions of Europe. Cardiovascular disease is the leading cause of premature mortality and Disability-Adjusted Life Years in Europe, accounting for 49% of all deaths; as such, this subject is of critical importance to public health because annual healthcare expenditures for cardiovascular disease in the European Union amount to an estimated EUR 192 billion [1,31].

Hypertension is a significant risk factor, if not the most significant, for nearly all acquired cardiovascular diseases, such as coronary artery disease, left ventricular hypertrophy, valvular heart disease, cardiac arrhythmias, including atrial fibrillation, cerebral stroke, and renal failure [3]. The guidelines for hypertension were jointly issued by the European Society of Hypertension and the European Society of Cardiology in 2003, 2007, and 2013, respectively [32]. As an illustration, The Hypertension Guidelines in 2003 were the most-cited article in the medical literature during 2003 and 2004, and the Guidelines in 2007 and 2013 have also received substantial citations [33]. The hypertension guidelines have provided an exhaustive synopsis of the correlation between hypertension and the risk of cardiovascular, cerebral, and renal complications. Unfortunately, this overview has been updated and modified marginally since 2013 [3].

In numerous observational studies, the associations between hypertension and cardiovascular mortality have been examined [34]. There exists an independent and continuous correlation between office blood pressure and the incidence of various cardiovascular events, including but not limited to stroke, myocardial infarction, sudden mortality, heart failure, and peripheral arterial disease [4]. This has been demonstrated across all age categories and ethnicities. The correlation with blood pressure spans from elevated levels of blood pressure to comparatively low values, specifically 70–75 mmHg for diastolic blood pressure and 110–115 mmHg for systolic blood pressure [3,8]. There is also a consistent correlation between out-of-office blood pressures obtained through 24 h ambulatory blood pressure monitoring in a residential setting and cardiovascular events. When blood pressure is high, metabolic risk factors, such as lipid abnormalities, glucose intolerance, and type 2 diabetes, are more prevalent than when it is low. The concurrent prevalence of these cardiovascular risk factors modifies the relationship between blood pressure and cardiovascular morbidity and mortality [3].

A class of anti-hypertensive drugs that has been used for half a century is renin–angiotensin–aldosterone system (RAAS) blockers. These drugs have been demonstrated to decrease cardiovascular events and slow the progression of chronic kidney disease [35,36]. A more novel approach that is in a clinical trial is to activate the angiotensin-converting enzyme 2 (ACE2)/Angiotensin-(1-7)/Mas receptor (MasR) pathway. The ACE2/Angiotensin-(1-7)/MasR axis acts as a counter-regulatory mechanism in the RAAS, opposing the actions of the classical ACE/Angiotensin II/Angiotensin Type 1 (AT1) receptor pathway. Activation of this axis has been demonstrated to produce beneficial cardiovascular effects. Angiotensin-(1-7) induces the release of nitric oxide from endothelial cells, which is crucial for maintaining healthy blood pressure levels [37,38]. Thus, therapies that target the angiotensin-converting enzyme 2 (ACE2)/Angiotensin-(1-7)/Mas receptor (MasR) pathway to prevent cardiovascular events and slow the progression of chronic kidney disease appear to be on the horizon.

Sodium–glucose cotransporter 2 (SGLT2) inhibitors were initially approved as an anti-diabetic treatment; however, their cardiovascular actions are gaining a great deal of attention [39]. Flozins represent the major class of SGLT2 inhibitors that act on the proximal tubule to increase glucose excretion, which results in diuresis, weight loss, and lowering of blood glucose levels. The diuretic action for flozins is known to help decrease hypertension and acute cardiac failure [40]. In addition, flozins slow the decline of the glomerular filtration rate, which prevents chronic kidney disease progression [39]. Overall, their cardiovascular and renal protective actions are due to long-term treatment with flozins. Additional anti-hypertensive therapies that reduce cardiovascular morbidity and mortality need to be developed.

## 5. Pathophysiology and Treatment

**Figure 1:**
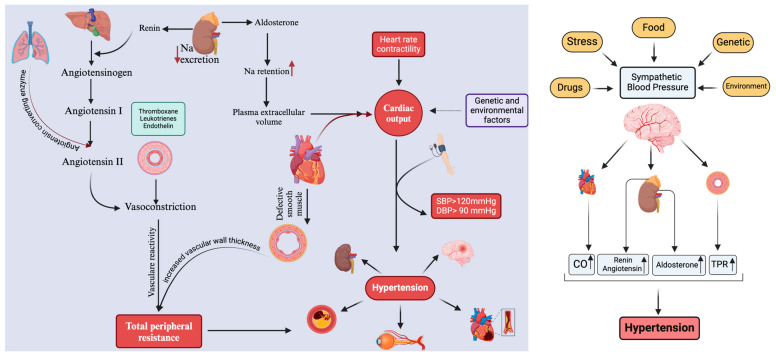
Hypertension considerations in different organs; high blood pressure affects all other organs of the body by affecting the cardiovascular and sympathetic nervous system. **Left Panel:** Major mechanisms controlling blood pressure are the renin–angiotensin–aldosterone system, kidney sodium retention, vasoconstriction and total peripheral resistance, and heart contractility and cardiac output. Hypertension can damage major organs, such as the kidney, brain, arteries, eyes, and heart. **Right Panel:** Environmental and genetic factors lead to increased sympathetic activity, which act on the kidneys, the adrenal gland, and the heart to increase blood pressure, resulting in hypertension. Created with BioRender.com.

Much remains to be learned regarding the pathophysiology of hypertension. An elevated blood pressure is attributed to an underlying renal or adrenal disease in a minority of patients (2% to 5%) [41,42]. The remainder, nevertheless, demonstrate “essential hypertension” due to the absence of a definitive, asymptomatic etiology. The maintenance of normal blood pressure is influenced by various physiological mechanisms, and disruptions in these mechanisms could potentially contribute to the onset of essential hypertension [43]. There are likely numerous interconnected factors at play in hypertensive patients’ elevated blood pressure, and the relative importance of these factors varies among individuals [44]. The renin–angiotensin system, the sympathetic nervous system, sodium intake, obesity, and insulin resistance are a few of the variables that have been the subject of extensive research. Other factors, such as genetics, endothelial dysfunction (indicated by alterations in endothelin and nitric oxide), low birth weight and intrauterine nutrition, and neurovascular anomalies, have been investigated over the years [45,46] (Figure 1).

The balance between cardiac output and peripheral vascular resistance maintains normal blood pressure. Most essential hypertensive patients have normal cardiac output but high peripheral resistance. Small resistance arteries with smooth muscle cells determine peripheral resistance [47]. Calcium channel blockers are an effective means to decrease peripheral resistance and lower blood pressure. Drugs that block calcium channels cause the smooth muscle cells to dilate small resistance arteries by decreasing the intracellular calcium concentration [48]. On the other hand, elevated angiotensin levels and prolonged smooth muscle contraction can thicken the arterial wall, causing an irreversible increase in peripheral resistance [49]. Primary hypertension may be due to sympathetic overactivity and higher cardiac output, not peripheral resistance [50]. The subsequent increase in peripheral arterial resistance may compensate for the elevated pressure to maintain capillary bed pressure and maintain cellular homeostasis [51,52].

Renin–angiotensin may be the most essential endocrine system for blood pressure management. Renin is released by the renal juxtaglomerular apparatus in response to low glomerular perfusion or reduced salt intake [53]. It is also released after stimulation of the sympathetic nervous system. Renin converts the renin substrate angiotensinogen to physiologically inactive angiotensin I, which is rapidly converted to angiotensin II by angiotensin-converting enzyme (ACE) in the lungs. Angiotensin II increases blood pressure by constricting blood vessels [53,54]. Drugs that target the RAAS play an important role in the management of high blood pressure and various cardiovascular and renal conditions. These include angiotensin-converting enzyme inhibitors (ACE inhibitors), such as enalapril, lisinopril, and ramipril, AT1 receptor blockers (ARBs), such as losartan, valsartan, and candesartan, and direct renin inhibitors (DRIs), a newer class of drugs [55].

In addition, it increases the secretion of aldosterone from the zona glomerulosa of the adrenal gland, which increases blood pressure due to sodium and water retention. The circulating renin–angiotensin system is not thought to directly increase blood pressure in essential hypertension. Many people with high blood pressure, especially the elderly and black people, have low levels of renin and angiotensin II, making renin–angiotensin system blockers ineffective. However, the “local” renin–angiotensin endocrine or paracrine systems that control blood pressure are becoming more apparent. Local renin systems are found in the kidney, the heart, and the arteries. They can control regional blood flow and, in the kidneys, control sodium excretion [53,54]. Activation of the RAAS is caused by juxtaglomerular cells in the kidneys releasing renin in response to diminished renal blood flow, low sodium levels, or sympathetic stimulation. Renin breaks angiotensinogen, a precursor peptide produced in the liver, into angiotensin I, which ACE converts to angiotensin II. Angiotensin II raises blood pressure by constricting blood vessels and increasing aldosterone levels. AT1 and angiotensin type 2 (AT2) receptors are the primary targets for angiotensin II. In pathological circumstances, the AT1 receptor predominates, causing vasoconstriction, increased kidney sodium reabsorption, adrenal gland aldosterone secretion, and sympathetic nervous system activation. These actions raise the blood volume and systemic vascular resistance and increase the heart rate. Angiotensin II and aldosterone establish a feedback loop that boosts vascular responsiveness and maintains high blood pressure [56].

Stenosis of the renal artery results in renovascular hypertension. This type of hypertension affects people of all ages. The underlying mechanism in renovascular hypertension involves activation of the RAAS pathway due to decreased perfusion to the kidney. Atherosclerosis leading to renal artery stenosis and hypertension is mostly seen in adults greater than 65 years old [57]. Interestingly, greater than 25% of all adults who die of cardiovascular disease have renal artery stenosis [40]. Anti-hypertensive drugs in combination with antiplatelet drugs and statins remain the foundation for the management of renovascular hypertension patients. Unfortunately, renovascular hypertension can be become difficult to control in elderly patients as renal function declines over time [40]. Percutaneous angioplasty is a treatment option that is used for renovascular hypertension due to fibromuscular dysplasia and for patients with atherosclerotic renal artery stenosis that is not controlled with medications [57].

The autonomic nervous system helps regulate blood pressure through actions on cardiac output and total peripheral resistance. Arterial smooth muscle cell constriction and increased cardiac output can be caused by stimulation of the sympathetic nervous system. It also causes short-term blood pressure fluctuations caused by stress and exercise. Limited data suggest that epinephrine and norepinephrine play a role in hypertension. However, their effects are significant because sympathetic nervous system blockers are an effective anti-hypertensive that lower blood pressure in hypertension [58].

Besides arteriolar smooth muscle cells, endothelial cells produce potent vasoactive factors, including nitric oxide and endothelin, which regulate the cardiovascular system. Essential hypertension is associated with endothelial dysfunction. To reduce the consequences of hypertension, modulation of endothelial function is an interesting therapeutic approach [59]. Nitric oxide is a major endothelial factor that impacts arteriolar function. Vasodilation occurs when the arterial and venous endothelium produces an endothelial-derived relaxing agent, now known as nitric oxide, which diffuses through the arterial wall into the smooth muscle [60]. Clinically effective anti-hypertensive therapy restores nitric oxide production but not endothelium-dependent vasorelaxation or agonist response. This suggests that endothelial damage is early and irreversible after the onset of hypertension [59].

Many other vasoactive systems and mechanisms regulate sodium transport and vascular tone to maintain normal blood pressure. However, their role in essential hypertension is unclear [61]. ACE inactivates bradykinin, a potent vasodilator. By preventing the inactivation of bradykinin, ACE inhibitors not only lower angiotensin II levels but also increase levels of the vasodilator bradykinin. Endothelin, a recently discovered vascular endothelial vasoconstrictor, can increase blood pressure in a salt-sensitive manner [62]. It also activates local renin–angiotensin systems. Heart atria release atrial natriuretic peptide in response to increased blood volume. As a natural diuretic, it increases the excretion of salt and water by the kidneys. A defective natriuretic peptide system can cause fluid retention, an increase in blood volume and cardiac output, and hypertension [63]. Ouabain, a naturally occurring steroid-like molecule, can interfere with cellular sodium and calcium transport and cause vasoconstriction [59]. Through its interaction with calcium transport and sodium transport across the vascular smooth muscle cell walls, this vasoconstriction can increase blood pressure.

Blood vessels, the heart, kidneys, and the central nervous system all influence blood pressure. Organ damage also occurs during hypertension. Heart damage is a major pathology that results from prolonged hypertension. Left ventricular hypertrophy with hypertension prevents diastole relaxation. Instead of the normal decrease in ventricular pressure, left atrial pressure increases ventricular input, especially during exercise, creating a suction effect. This can increase pulmonary capillary pressure enough to cause congestion [64]. Atrial fibrillation can result from high atrial pressure, and hypertrophied ventricles depending on atrial systole can lose atrial conduction, reduce stroke volume, and cause pulmonary edema. Exercise-induced subendocardial ischemia can potentially “magnify” hypertrophic abnormalities of myocardial diastolic relaxation [65].

Immune cells also play a crucial role in hypertension and causing the organ damage observed in hypertension. There is a strong, automatic, nerve-related, anti-inflammatory route that is always active and blocks the activation of a protein called NF-κB in immune cells [66,67]. This system does not function properly in individuals with hypertension. Extensive research has conclusively shown the significant involvement of the adaptive immune system and lymphocytes in the development of hypertension [68,69]. Understanding the role and method of action of the innate immune system in hypertension is highly significant, as it primes and activates the adaptive immune system. In the most common hypertension types, it is important to determine the impact of certain immune cell populations, such as monocytes, macrophages, natural killer (NK) cells, and T helper cells (Th17) cells, on blood pressure regulation and damage to organs [70]. Cell sorting, targeted removal of specific immune cell populations, and transfer of cells can effectively handle these problems, which will have significant implications for translation into practical applications [71].

Lastly, birth weight may affect adult blood pressure, according to growing data. Babies born small are more prone to hypertension in adolescence and adulthood. Small infants are more prone to metabolic abnormalities, such as insulin resistance, diabetes mellitus, hyperlipidemia, and abdominal obesity, which can lead to hypertension and cardiovascular disease (the “Barker hypothesis”) [72]. Insulin resistance can increase cardiovascular disease in underweight individuals. However, genetics could influence Barker’s hypothesis. Younger babies born to mothers with hypertension develop hypertension and higher than average blood pressure. In a modern “healthy” society, similarities in maternal and child blood pressure are likely to be heritable and unrelated to intrauterine malnutrition [73].

**Figure 2:**
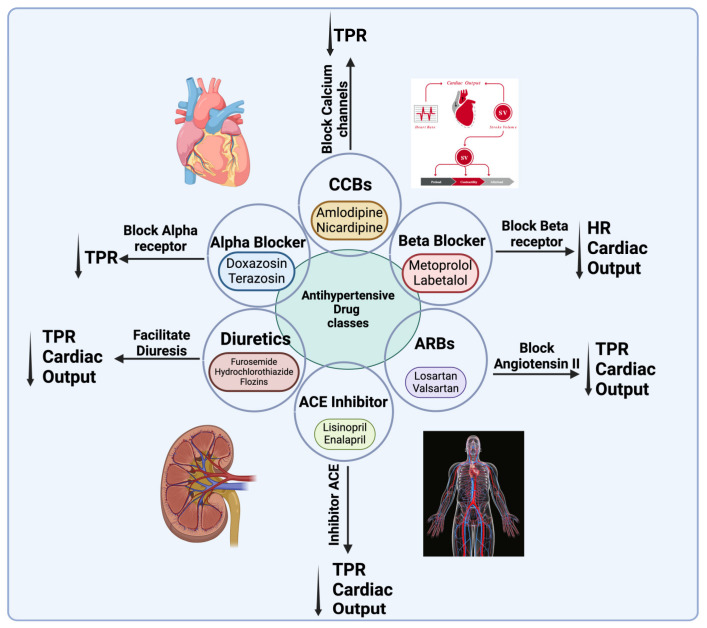
Classes of anti-hypertensive drugs affect blood pressure reduction through different pathways. There are six classes of anti-hypertensive drugs: calcium channel blockers (CCBs), beta-blockers, angiotensin receptor blockers (ARBs), angiotensin-converting enzyme inhibitors (ACE), diuretics, and alpha blockers. Anti-hypertensive drugs act on the heart, small resistance arteries, and kidney to decrease total peripheral resistance (TPR) and cardiac output. Created with BioRender.com.

There are many types of drugs used to control and treat hypertension, which are classified according to their performance in different categories (Figure 2). Diuretics, commonly known as “water pills”, are one category of anti-hypertensive drugs. This class of anti-hypertensive drugs help eliminate excess salt and water, thereby reducing blood volume and lowering blood pressure [74]. They are often used as first-line drugs, especially in adults, older people, and people with conditions like heart failure or kidney disease. These include hydrochlorothiazide, which is often the first choice among diuretics and even effective in lower doses. Furosemide (Lasix), which is a ring diuretic, is mainly for patients with fluid retention or heart failure cases. Aldosterone antagonists, such as spironolactone, a potassium-sparing diuretic that helps prevent potassium loss, is often combined with thiazide diuretics to counter hypokalemia. SGLT2 inhibitors represent a newer class of diuretics that have anti-diabetic and anti-hypertensive actions [39]. However, it cannot be denied that diuretics can cause side effects, including recurrent urine or polyuria, electrolyte imbalances, and increased uric acid levels that can exacerbate gout [75].

The first-in-line RAAS anti-hypertensive drugs are ACE inhibitors that inhibit the conversion of angiotensin I to angiotensin II. They are especially recommended for patients with diabetes or coronary artery disease. These include lisinopril (Prinivil, Zestril), which has been widely welcomed for its effectiveness and the relatively low side effects reported. There is also enalapril (Vasotec), which is an effective drug, but it has an increased incidence of cough compared to other ACE inhibitors. If these side effects are associated with increased potassium levels and kidney dysfunction, it may require regular monitoring [76]. ARBs act similarly to ACE inhibitors but block the function of angiotensin II instead of lowering its levels. They are especially useful in patients who cannot withstand ACE inhibitors due to the coughing side effects. Losartan (Cozaar) is an ARB that is often prescribed due to its rapid and acceptable effectiveness and minimum side effects. Also, Valsartan (Diovan), which is a drug like Losartan, can effectively control blood pressure, is well-tolerated in the body, and has minimal side effects [77].

Drugs that act to decrease sympathetic activity and block calcium channels are also effective anti-hypertensives. Beta-blockers reduce blood pressure by reducing the heart rate and heart work. These drugs are often prescribed for patients with a history of heart attacks or specific types of migraines. Athenolol (tennormine) and metopressor (lopressor) fall into this group. Beta-blockers are also more effective in young adults with hypertension because blood pressure is more dependent on cardiac output at that age. Calcium channel blockers, as their name implies, prevent calcium from entering into cardiac and vascular smooth cells. They are especially effective for patients with angina or specific heart rhythm. Amlodipine (Norvasc) and Diltizem (Cardizem) are among these drugs [78].

Recent hypertension management guidelines were published for the European Society of Hypertension in 2023 and the European Society of Cardiology Congress in 2024 [79,80]. Lifestyle management remains one of the most important steps to control blood pressure. A healthy diet, lower salt consumption, lower alcohol consumption, and regular moderate to intense aerobic exercise are needed. First-in-line drugs are RAAS inhibitors and diuretics or their combination with beta-blockers as needed for specific indications. Hypertension treatment objectives are to lower blood pressure below 140/80 mmHg, and, if possible, to reach values <130/80 mmHg. Attaining these treatment goals should lower the incidence of chronic kidney disease, heart disease, and cardiovascular morbidity and mortality.

## 6. Hypertension in Children and Adolescents

There is a lack of direct evidence that establishes a correlation between the screening of children and adolescents for hypertension and the prevention or delay of cardiovascular outcomes in adults, and the quality of indirect evidence is inconsistent [81]. The data that have been reported indicate that a significant number of children who exhibit elevated blood pressure during screening will not develop hypertension. There is also some evidence to suggest that hypertension in childhood is associated with hypertension in young adults or has low to moderate sensitivities and specificities for predicting adult hypertension [82]. Additionally, the correlation between childhood hypertension and carotid intima-media thickness and microalbuminuria in young adults is inconclusive, and there is a paucity of direct evidence regarding other intermediate or final health outcomes.

Even though hypertension is more prevalent among the elderly, there is a rising prevalence among youth. Nevertheless, the prevalence of hypertension diagnoses among youthful individuals who meet the diagnostic criteria is lower than that of middle-aged and older individuals [83]. Due to this, it may be advantageous to concentrate public health preventative initiatives on adolescents who are at an elevated risk of developing hypertension. Additionally, the management of hypertension is simpler and less time-consuming for youthful individuals than it is for older individuals. In young adults, prehypertension is more common than hypertension, which is a major precursor to hypertension and cardiovascular disease in later life [84]. When prehypertension is diagnosed early, it may be reduced with lifestyle changes. Not many studies have been performed on hypertension in young adults, and this group is usually ignored. Reports have shown that excessive sodium intake, obesity, dyslipidemia, and smoking are more common among young people with hypertension than in the general population [85].

Clinical trials have been conducted even though the incidence of hypertension is lowest in young individuals compared to middle-aged adults and the elderly. There have been several medium- and short-term trials conducted to evaluate the effectiveness of anti-hypertensive drugs in children and adolescents [86] (Figure 2). Each trial focused on a distinct agent. The results of interventions for the treatment of elevated blood pressure that involve lifestyle interventions alone or in combination with an anti-hypertensive medication are inconsistent. Increasing physical education at school was effective in reducing blood pressure [87]. The effects of an anti-hypertensive combined with a complex lifestyle program (the ADAPT program) were not sustained. Furthermore, a low-sodium diet combined with personalized support was only effective in girls [87]. The studies that have been conducted on hypertension in children and adolescents with normal body mass index (BMI) are limited, and for more certainty, we need to conduct more studies on this group. The screening of minors for hypertension has the potential to move the management of hypertension to younger age groups and potentially reduce the risk of cardiovascular disease in adults in the future [88].

## 7. Hypertension in Middle Age and Old Age

Hypertension rises in middle-aged adults and has been linked to sleep quality. In the adjusted Cox models, the total population exhibited a higher risk of developing hypertension due to poor sleep quality. Regarding the habitual sleep efficiency of the <65%–75% group, mild and moderate sleep disturbance and poor subjective sleep quality all increased. This elevates their susceptibility to hypertension in comparison to their classmates who are developing it. The youthful and middle-aged population were at a higher risk of developing hypertension due to poor sleep quality based on age stratification [89,90].

The prevalence of hypertension increases greatly between middle-age and old-age adults. There is also an accelerated aging of populations worldwide. By 2050, approximately 2 billion individuals worldwide will be 60 years or older, with 400 million individuals aged 80 years or older. Most elderly individuals succumb to non-communicable diseases, including hypertension, heart disease, cancer, and diabetes, rather than infectious and parasitic diseases. Hypertension is one of the leading causes of death worldwide. According to World Health Organization (WHO) reports, high blood pressure is responsible for approximately 12.8% of all deaths or 7.5 million deaths per year [91,92].

Aging and hypertension are closely related, and their combined effects can have a significant impact on cognitive function. Hypertension in the elderly usually occurs with normal or even low cardiac output and a combination of factors, such as a small component of increased peripheral vascular resistance or a significant increase in the stiffness of the large artery. As people age, their arteries become less compliant, a condition known as arterial stiffness. This increase in arterial stiffness is often associated with abnormally hypertension levels. Arterial stiffness with functional and structural mechanisms is different in old people compared to young people [93]. Atherosclerotic renovascular hypertension is the most prevalent hypertension in the elderly [40]. Moreover, high blood pressure and increased arterial stiffness, when associated with aging, are associated with an increased risk of cognitive decline and the development of dementia [94,95]. Some of the mechanisms that are potentially enhanced by hypertension in old age include increased oxidative stress and neuroinflammation in the brain, which can lead to nerve damage and death. The combination of hypertension and aging appears to have a synergistic effect on cognitive decline, with older individuals with hypertension showing more pronounced impairments in learning, memory, and other cognitive domains compared to their younger counterparts or individuals without hypertension [96].

## 8. Hypertension in Economic and Social Situations

Lower socioeconomic status, such as lower income, education, and occupational levels, is consistently associated with higher risk and prevalence of hypertension in various populations [97]. Reports have shown that the probability of hypertension in low-income families is 1.35 times higher than in families with a higher annual household income. Also, the prevalence of high blood pressure is greater among people with no education compared to people with higher education. Likewise, lower occupational status, such as unemployment or inability to work, is associated with increased prevalence of hypertension [98].

In addition to this information, it should be noted that limited access to healthcare services and anti-hypertensive drugs is an important obstacle for effective blood pressure control and hypertension management. Inadequate access to care is recognized as a key factor affecting the treatment and control of hypertension. Poor blood pressure management in youth is often associated with limited access to care and increases the likelihood of future cardiovascular events. A temporary decrease in access to healthcare is also associated with a long-term increase in the rate of uncontrolled hypertension [20]. Socioeconomic inequalities, particularly in wealth, are strong independent predictors of hypertension, particularly among older populations. As the quartile of wealth decreases, the risk of developing hypertension increases by 17–28% in women and 17–20% in men [99,100].

## 9. Body Mass Index and Hypertension

An elevated BMI is strongly associated with an increased risk of hypertension. Reports have shown that for every 3 kg/m^2^ increase in BMI, the risk of developing hypertension increases by about 30%. BMI has a positive correlation with systolic blood pressure and diastolic blood pressure. This relationship is true across all BMI ranges, including overweight and obese categories [101]. People who are overweight or obese have a significantly higher prevalence of hypertension compared to people who have a normal weight [102]. Observations have shown that the effect of BMI on blood pressure is dose-dependent; as the BMI level increases, the probability of hypertension increases. Previous studies have shown that 45% of participants with a normal BMI had hypertension, compared to 67% of overweight participants and 79–87% of obese participants [103,104]. The potential association between BMI and hypertension risk is influenced by various factors, such as age, gender, and ethnicity. Therefore, maintaining a healthy BMI is recognized as a key strategy for preventing and managing hypertension [105].

## 10. Lifestyle Factors and Hypertension

**Figure 3:**
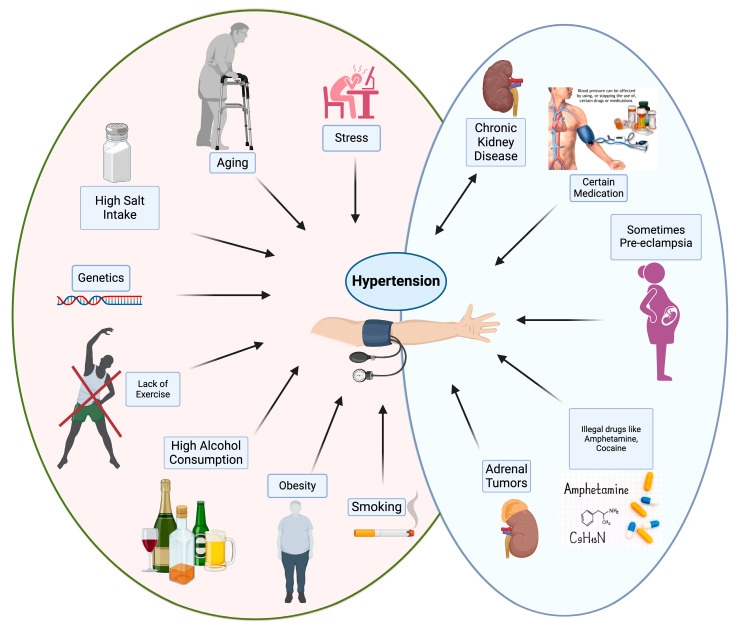
The specific living conditions of individuals influence the occurrence and intensity of hypertension. Factors contributing to hypertension, including stress, high salt intake, and smoking, appear on the left side in red. Other factors contributing to hypertension are provided on the right side in blue, which includes chronic kidney disease, medications, pregnancy, and adrenal tumors. Created with BioRender.com.

A diet rich in sodium and low in potassium is one of the main factors behind the projected increase in hypertension worldwide [11]. Physical inactivity is also one of the most important risk factors for hypertension. Being overweight or obese dramatically increases the risk of heart disease and its risk factors, including hypertension. On the other hand, family history and genetic predisposition are important risk factors for hypertension. Some rare genetic disorders, such as hyperaldosteronism, Liddle’s syndrome, and paraganglioma, can also cause hypertension [106]. Additionally, the renin–angiotensin–aldosterone system and the genes affecting the vascular endothelium play an important role in blood pressure and should be taken into consideration. Finally, chronic psychosocial stress is associated with increased risk of hypertension. Stress hormones may play a role in hypertension, although more research is needed to fully understand the relationship [107]. See Figure 3.

In today’s life, hypertension is a common and serious disease that affects a significant part of the world’s population. Hypertension is a major risk factor for various life-threatening cardiovascular diseases and cerebrovascular diseases, especially heart attack, stroke, kidney failure, and vision problems. Untreated hypertension can lead to severe and potentially fatal complications, making it a critical public health issue that requires comprehensive research and effective management strategies [16,108]. Uncontrolled blood pressure has severe consequences, leading to a wide range of cardiovascular complications [109]. On the other hand, studies have shown that hypertension is related to cognitive decline, and there is a relationship between this disease and increased risk of transient ischemic attack, stroke, dementia, and mild cognitive impairment [110]. Lifestyle adaptation in combination with anti-hypertensive drugs are necessary to decrease these hypertension complications.

Digital health solutions, such as wearable devices and digital health apps, are transforming lifestyle adaptation and blood pressure management. These solutions can facilitate data-driven approaches to personalized care. However, ensuring equitable access to these technologies and evaluating their effectiveness is critical to avoid misallocation of resources. Today, the development of digital therapies is accelerating, which, if implemented properly, could improve the management of many diseases, including hypertension, and reduce the global burden of cardiovascular [111]. Importantly, digital health interventions are effective instruments for managing hypertension, particularly in underserved populations. Hypertension can be monitored, and patients’ engagement in their health can be improved using mobile applications and devices. Text message reminders and content are utilized by certain applications to enhance treatment regimens. The potential efficacy of these tools in managing hypertension is demonstrated by the evidence that they can result in a substantial reduction in systolic blood pressure over time [112].

## 11. Conclusions

Hypertension has a staggering economic burden on governments. Its annual costs are estimated at USD 79 billion in the United States alone [113]. These direct costs include medical costs, such as hospitalization and prescription drugs, as well as indirect costs, such as lost productivity and absenteeism. By improving the management and control of blood pressure, it is possible to significantly save healthcare costs and improve the overall health of the population [114]. Despite the societal need and severe consequences of hypertension, there are several research needs that require further study. One of the important areas of concern is the limited understanding of the pathophysiology of hypertension based on gender, because in most of the studies, the presence of women in the study groups is insufficient [115].

The existence of inequality in health is another suitable topic to study hypertension. These comparisons need more research, in addition to the issue of gender [116]. Certain racial and ethnic groups, such as non-Hispanic black adults, show a higher prevalence of hypertension and poorer control rates than other populations. Understanding the underlying causes of these disparities and implementing targeted interventions are essential to achieve more appropriate hypertension management for vulnerable communities [20].

Hypertension and co-morbidities, such as diabetes, also need further investigation. Treatment and control of these conditions require screening, patient education, and health centers [117]. Additionally, further investigation is needed to identify barriers and facilitators of hypertension control, particularly in low- and middle-income communities, where access to healthcare and resources may be limited. Emerging technologies and innovative therapeutic strategies offer promising ways to advance the management of hypertension. Digital health interventions, including self-monitoring and remote monitoring, have shown potential to improve blood pressure control [118]. In addition, new pharmacological and procedural therapies, such as renal denervation and baroreflex activation, offer new options for controlling and managing hypertension [119]. The promise of AT2 receptor agonist drugs to treat hypertension and other cardiovascular diseases could be on the horizon [120]. Excitingly, aprocitentan (TRYVIO), an endothelin (ET) receptor antagonist (ETRA), was recently approved for resistant hypertension [121]. The development of additional drugs that target specific hypertension types with precision are sure to be approved in the future.

Continued research on hypertension and investment in its management and related diseases is vital. By expanding our understanding of the underlying mechanisms, improving access to effective interventions, and taking advantage of technological advances, researchers and healthcare providers can work towards reducing the devastating impact of this condition.
